# Impact of Iodine Deficiency Trends on the Pathophysiology of Selected Populations

**DOI:** 10.1016/j.advnut.2025.100411

**Published:** 2025-04-15

**Authors:** Nicola De Maio, Fiore Carpenito, Alberto Carpenito, Francesco Turco, Giancarlo Landi, Margot De Marco

**Affiliations:** 1 Fibrosys srl, Academic Spin Off, University of Salerno, Baronissi, Italy; 2 A.O.S.G. Moscati Hospital, Endocrine Nutrition and Replacement Diseases U.O.S.D., Avellino, Italy; 3 ASL Avellino, Health district of Ariano Irpino District, Avellino, Italy; 4 Department of Medicine, Surgery and Dentistry “Scuola Medica Salernitana”, University of Salerno, Baronissi, Italy

Dear Editor:

In a very interesting article, Liang et al. [1] analyze global iodine deficiency (ID) trends from 1990 to 2021, with projections through 2050, by using the extreme gradient boosting (XGBoost) + Shapley additive explanations (SHAP) model. The authors comment on how the model effectively identifies key factors (age, sex, and year) to produce accurate predictions, and they envision future integration of additional variables, such as local socioeconomic factors, to improve the applicability of the model in specific regions.

As the authors underline, regional, and demographic characteristics must be considered to fully interpret the effects of ID reduction. In this regard, we would like to reflect on the impact of globalization and migratory phenomena, which introduces variability in genetic characteristics, dietary trends, and lifestyle in populations belonging to the same regional unit. We believe that the analysis of populations with a homogeneous genetic substrate and stable habitat over time can help to appreciate the extent of the direct effects of ID reduction on selected indicators.

In support of this point, we would like to briefly comment on our analysis carried out on a population with stable genetic characteristics and habitat. In 2021, we analyzed thyroid nodule incidence in a population of 200 adults residing in a small town of the Southern Italy macro area; habitat and population genetics can be considered invariant from 1990 to today. Over 65% of the studied subjects habitually consumed iodized salt. Thyroid nodules were observed in 40% of the participants. We compared these data with those we obtained in a study performed in 1993 of 45 adults of the same community and age range. Only <2% of them consumed iodized salt, but 86% of this population presented with thyroid nodules. Therefore, iodized salt intake appeared to be related to a highly significant reduction in the incidence of thyroid nodules (*P* < 0.0001 in a chi-squared analysis).

Such a homogeneous population does not reflect the complexity of the real world. However, we believe that data in this type of context can help to appreciate, using a direct indicator (goiter) of the most common pathology related to ID, the magnitude of the effect of the salt iodization policy in the absence of confounding elements resulting from population mixing and habitat changes.

## Conflict of interest

The authors report no conflicts of interest.

## References

[1] Liang D., Wang L., Zhong P., Lin J., Chen L., Chen Q. (2025). Perspective: Global burden of iodine deficiency: insights and projections to 2050 using XGBoost and SHAP. Adv. Nutr..

